# Therapeutic Effects of Oligonol, Acupuncture, and Quantum Light Therapy in Chronic Nonbacterial Prostatitis

**DOI:** 10.1155/2015/687196

**Published:** 2015-05-03

**Authors:** İlhan Öztekin, Hakan Akdere, Nuray Can, Tevfik Aktoz, Ersan Arda, Fatma Nesrin Turan

**Affiliations:** ^1^Departments of Anesthesiology & Algology, Medical Faculty, Trakya University, Balkan Campus, 22030 Edirne, Turkey; ^2^Department of Urology, Medical Faculty, Trakya University, Balkan Campus, 22030 Edirne, Turkey; ^3^Department of Pathology, Medical Faculty, Trakya University, Balkan Campus, 22030 Edirne, Turkey; ^4^Department of Biostatistics, Medical Faculty, Trakya University, Balkan Campus, 22030 Edirne, Turkey

## Abstract

This research aimed to compare anti-inflammatory effects of oligonol, acupuncture, and quantum light therapy in rat models of estrogen-induced prostatitis. Adult male Wistar albino rats were grouped as follows: Group I, control (*n* = 10); Group II, chronic prostatitis (*n* = 10); Group III, oligonol (*n* = 10); Group IV, acupuncture (*n* = 10); Group V, quantum (*n* = 10); Group VI, oligonol plus quantum (*n* = 10); Group VII, acupuncture plus oligonol (*n* = 10); Group VIII, quantum plus acupuncture (*n* = 10); and Group IX, acupuncture plus quantum plus oligonol (*n* = 10). Chronic prostatitis (CP) was induced by the administration of 17-beta-estradiol (E2) and dihydrotestosterone (DHT). Oligonol was given for 6 weeks at a dose of 60 mg/day. Acupuncture needles were inserted at CV 3/4 and bilaterally B 32/35 points with 1-hour manual stimulation. Quantum therapy was administered in 5-minute sessions three times weekly for 6 weeks. Lateral lobes of prostates were dissected for histopathologic evaluation. Although all of the treatment modalities tested in this study showed anti-inflammatory effects in the treatment of CP in male rats, a synergistic effect was observed for oligonol plus quantum light combination. Monotherapy with oligonol showed a superior anti-inflammatory efficacy as compared to quantum light and acupuncture monotherapies.

## 1. Introduction

Nearly 50% of all men experience prostatitis like symptoms at least once during their lifetime [1, 2]. The cause and pathogenesis of nonbacterial prostatitis is obscure, and its chronic course is associated with therapeutic challenges [1, 2]. Lewis and Wistar rats represent appropriate animal models for experimental CP studies due to the fact that their aging is associated with spontaneous development of nonbacterial prostatitis [3]. Administration of estradiol (E2) causes increased frequency and severity of prostatitis in adult male Wistar rats [4, 5]. Naslund et al. established the emergence of the same histological findings in spontaneous prostatitis and E2-induced prostatitis in Wistar rats. In a number of different studies, spontaneous nonbacterial prostatitis in rats has been shown to exhibit histological features that are very similar to those in human CP [6, 7].

Polyphenol hydroxyl group, a secondary metabolite substance present in green tea, inhibits inflammation upon being activated with the effect of several antioxidant enzymes. It has been shown to play an important potential role in the prevention of several degenerative conditions such as heart disease and cancer [8–10]. Shoskes et al. showed at least 25% improvement in the symptom scores in 67% of category III CP patients receiving 5-bioflavonoid quercetin. Oligonol, an oligomerized polyphenol (Amino Up Chemical Co., Sapporo, Japan), consists of high concentrations of lower oligomers such as monomers, dimers, trimers, and tetramers that are obtained through the depolymerization of catechin-type polyphenol [11]. In the study by Kim et al., oligonol has been found to be efficacious in the prevention of chronic abacterial prostatitis in rats [1].

Quantum light therapy, which is increasingly more commonly used, has its roots in the early 1990s. It can be defined as the collection of data sources, pathways, and methods based on the use of electromagnetic waves, quantum processes, and the informative wave characteristics of the organism. Its effects occur at a cellular and molecular level and they are dispersed throughout the organism via a chain reaction. The wide range of therapeutic effects of the quantum devices is explained based on their ability to allow a communication between electromagnetic information and proteometabolic processes, as well as a special concordance with the organism [12].

The following electromagnetic beams are chosen for use in quantum therapy devices: low intensity, coherent, and super pulsed laser beams with a wavelength of 905 nanometer; wideband pulsed infrared beams with wavelengths between 890 and 960 nanometer; pulsed visible red light with wavelengths between 640 and 740 nanometer; constant magnetic field with an intensity of 35 millitesla [12].

Super pulsed coherent laser beams penetrate deep into tissue (12-13 cm) with strong stimulant effects on blood circulation, cell membrane, and intracellular metabolism. It also activates neurohumoral factors and immunocompetent systems and regulates the endocrine system. Low level laser therapy (LLLT) has a positive effect on the inflammatory process and tissue regeneration [12–17].

The pulsed noncoherent infrared beams possess a wider spectral band with lower penetration abilities, affecting a variety of reflexogen regions, and exhibit a strong regulatory effect on the healthy central nervous and vegetative nervous systems [12].

Pulsed red light, with even lower penetration characteristics than with infrared light, reduces the intensity of inflammation in articular structures, particularly in porous tissues [12, 18].

A constant magnetic field has been designed to serve as an energy barrier against environmental noxious factors including air. It facilitates the penetration of laser beams, allowing the reduction of radiation required to achieve the same level of effect with laser monotherapy [12, 19].

Acupuncture is based on the insertion of sterile needles in acupoints according to certain canal and meridian systems, which were originally defined by the early practitioners of the traditional Chinese medicine (TCM). The needles are rotated manually and stimulated electrically or thermally [20, 21]. Although physiological mechanisms of acupuncture are unknown, several hypotheses exist. For example, acupuncture treatment is thought to regulate the pain control mechanisms within the central nervous system (CNS) through the release of specific neurotransmitters, such as endorphins [21–24].

Acupuncture is generally used for the alleviation of chronic painful conditions [25–29]. Beneficial effects on urinary symptoms and quality of life in patients with chronic prostatitis/chronic pelvic pain syndrome (CP/CPPS) have been reported [30].

The aim of this study was to compare anti-inflammatory effects of oligonol, acupuncture, and quantum light therapy (magnetic infrared laser) in rat models of estrogen-induced nonbacterial prostatitis.

## 2. Materials and Methods

### 2.1. Chemical Reactants

Oligonol manufactured by Quality of Life Labs (NY, USA) by polymerization of polyphenols found in lychee fruit and green tea extract was used in the study. 17-beta-estradiol (E2), testosterone (T), dihydrotestosterone (DHT), and hematoxylin and eosin were provided by Sigma Aldrich (Steinheim, Germany).

### 2.2. Animals and Treatment

The adult male Wistar albino rats (age > 3 months) were obtained from the Animal Experiment Laboratory, Trakya University (Edirne, Turkey). They were accommodated under controlled temperature and humidity conditions with successive 12-hour cycles of darkness and light. The Local Ethics Committee for Animal Experimentation, Trakya University, approved the treatment protocols. In order to induce lateral prostatic lobe inflammation, a modified estrogen protocol originally proposed by Robinette [31] and Naslund et al. [4] was used. A total of 9 treatment groups with 10 rats in each group were established as follows: Group I, normal controls; Group II, chronic prostatitis; Group III, oligonol; Group IV, acupuncture; Group V, quantum; Group VI, oligonol plus quantum; Group VII, acupuncture plus oligonol; Group VIII, quantum plus acupuncture; Group IX, acupuncture plus quantum plus oligonol. The experimental protocol is shown in Table 1. The normal control group received no treatment. Subcutaneous E2 administration was performed for 4 weeks in animals in the chronic prostatitis group and in other treatment groups, with the additional DHT for two weeks after day 15. E2 and DHT were dissolved in sesame oil and were given subcutaneously at a dose of 250 microgram/kg/day. In oligonol groups, oligonol at a dose of 60 mg/kg/day was given for 6 weeks via oral feeding tube after being diluted with drinking water. The same oligonol protocol was used in combination treatments involving oligonol use. Before each session in the acupuncture groups, ketamine (50 mg/kg, i.m.) and xylazine (5 mg/kg, i.m.) were given for anesthesia. Steel needles (0.20 × 13 mm) produced by Hua Long Co. (China) were applied for a total duration of 6 weeks at conceptual vessels (CV) 3 and 4 and bilaterally urinary bladder (Bl) 32 and 34 points under the guidance of rat anatomy atlas [32] and atlas of acupuncture [33] by an acupuncturist for three days a week, with 1-hour daily sessions and manual stimulation every 10 minutes. The same acupuncture protocol was used in combination groups involving acupuncture treatment. CV 3 and 4 points are on the anterior midline of the abdomen, 4/5 and 3/5 of the way down from the umbilicus to the superior edge of the pubic bone. B 32 point is on the region of the sacrum, on the 2nd sacral foramen. B 34 point is on the region of the sacrum, medial, on the 4th sacral foramen [33].

In the quantum therapy groups, a RIKTA-04/4 Magnetic Infrared Laser Treatment device (JSC MILTA-PKP GIT, Moscow, Russia) was used together with the above mentioned anesthesia protocol under the guidance of a rat anatomy atlas [32] in the following manner with 5-minute daily sessions for 3 days a week for a total duration of 6 weeks: through an emitter with a laser pulse strength of 12 W and active application area of 4 cm^2^ to suprapubic, urinary bladder, anterior penile, and femoral artery (bilateral) regions and through a Douche Emitter with a laser pulse strength of 30 W and active circular application area of 20 cm^2^ to mid-perineum region between testicles and anus (including prostate area) (Table 2). The same protocol was also used in combination treatments involving quantum therapy.

### 2.3. Body/Prostate Weight and Histopathology

**Figure 1:**
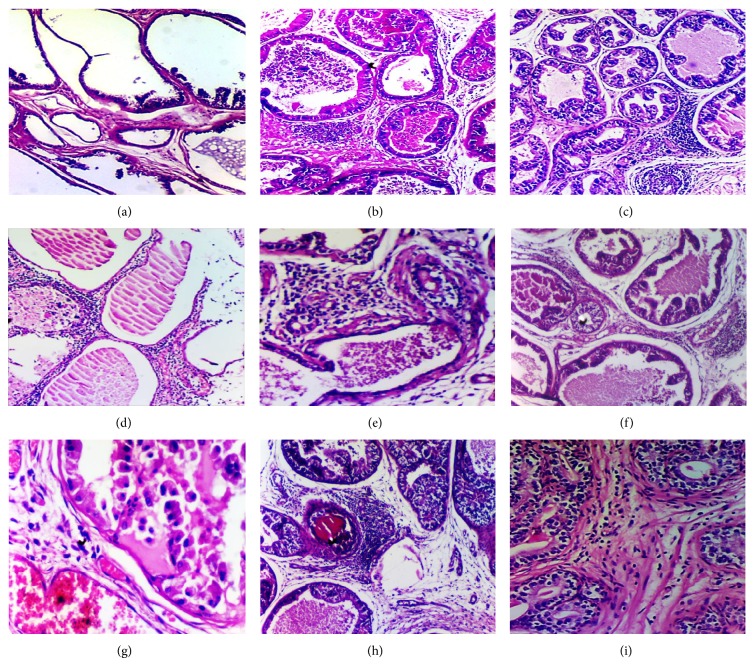
Histological views of prostatic inflammation in all groups. Normal controls; Group I, (hematoxylin and eosin, ×100) (a); Group II, chronic prostatitis (hematoxylin and eosin, ×100) (b); Group III, oligonol (hematoxylin and eosin, ×100) (c); Group IV, acupuncture (hematoxylin and eosin, ×100) (d); Group V, quantum (hematoxylin and eosin, ×200) (e); Group VI, oligonol plus quantum (hematoxylin and eosin, ×100) (f); Group VII, acupuncture plus oligonol (hematoxylin and eosin, ×200) (g); Group VIII, quantum plus acupuncture (hematoxylin and eosin, ×100) (h); Group IX, acupuncture plus quantum plus oligonol (hematoxylin and eosin, ×200) (i).

**Figure 2:**
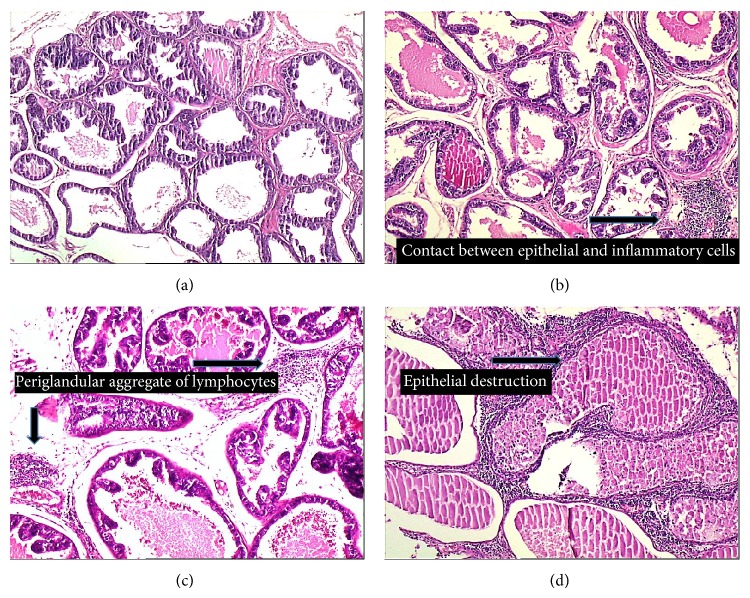
Histological views of prostatic inflammation. Control group without any inflammation, grade 0 (hematoxylin and eosin, ×100) (a). Some contact between epithelial cells and inflammatory cells (black arrow), grade 1 (hematoxylin and eosin, ×100) (b). Periglandular infiltrates adjacent to partially destroyed epithelium (black arrows), grade 2 (hematoxylin and eosin, ×100) (c). The inflammation was intensive (black arrow) and the number of inflamed acini was more than 25%, grade 3 (hematoxylin and eosin, ×100) (d).

Body weight measurements were performed in all groups at days 1, 20, and 40. After the prostate gland weight was measured without bladder or seminal vesicles, both lateral lobes of prostates were dissected and used for histopathologic evaluation. Samples were fixed in neutral 10% formalin solution for 24 hours at room temperature, dehydrated in ethanol, cleared in xylene, and embedded in paraffin. Hematoxylin and eosin stained 4 micron sections were obtained for histological examination. Double pathologist blinded assessments for pathological evaluations were performed. Prostatitis was considered if inflammatory cell infiltration in epithelial cells of single acini was present. The inflammation was scored according to its intensity and distribution through the prostatic tissue (Figure 1). In this study, a four-tiered scale system applied by Bernoulli et al. was used: grade 0 = no contact between inflammatory cells and epithelium; grade 1 = some contact; grade 2 = periglandular infiltrates adjacent to partially destroyed epithelium; and grade 3 = the number of these acini was more than 25% (Figure 2). The numbers of inflamed acini were counted for the entire prostate area using the same sample sections.

### 2.4. Statistical Analyses

All data were presented as mean ± SD. The distributions of all continuous variables for normal distribution were tested using the Kolmogorov-Smirnov one sample test. The comparison between and within groups was performed using repeated measures of ANOVA; the comparison of the groups was performed using one way ANOVA and binary (post hoc) comparisons Tukey b and Dunnett T3 tests. The comparison within groups was evaluated using paired *t*-test. Analyses were performed using the SPSS 20.0 Statistical Package Program.  *P*  values < 0.05 were considered statistically significant.

## 3. Results 

The total numbers of rats remaining in the study groups at the end of the 6-week experimental protocol are shown in Table 3.

### 3.1. Body Weight and Prostate Weight

**Figure 3:**
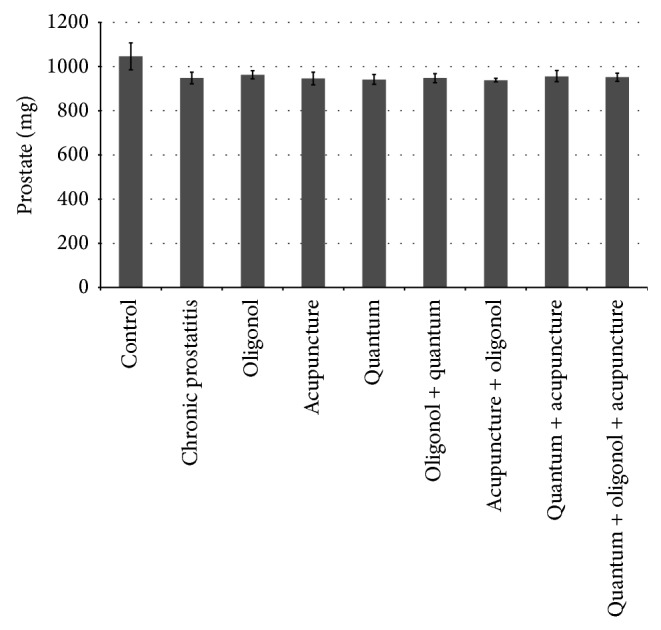
Prostate weight groups on day 40.

An increase in body weight was observed in all groups, as evidenced by the body weight measurements at days 1, 20, and 40. Despite the absence of a significant difference between the groups in body weight from days 1 to 20, a significant increase at day 40 was noted in Group I compared to Groups VI, VII, and IX (*P* = 0.032, *P* = 0.007, and *P* = 0.009, resp.). In oligonol groups, significantly fewer increases in body weight at day 40 were found as compared to the normal control group (Table 3). However, no significant differences in terms of body weight increase were noted between the normal control group and others. The normal control group showed significantly higher prostate weight compared to the other groups (*P* = 0.0001) (*P* = 0.016, 0.017, 0.009, 0.021, 0.047, 0.017, 0.0.010, and 0.028, resp.) (Table 3) (Figure 3). A total of 13 rats in all groups died after anaesthesia (Table 3). Side effects, such as lethargy and mortality, were observed in one rat in response to oligonol.

### 3.2. Histopathology

In the normal control group, there was almost a normal appearance (median prostatitis score: 1) of the glandular epithelium and stroma with a few leukocyte infiltrations into the lumina and stroma that refers to a grade between 0 and 1 in all rats. Extensive infiltrations of inflammatory cells in the lumina, mononuclear cells in the stroma of the gland, and epithelial degeneration were observed in Group II suggesting CP. Of the 10 rats in this group, 4 rats showed grade 3 and 3 showed grade 2 inflammations.

According to the prostatitis score, there were significant differences between the groups (*P* = 0.023): between Group I and Groups II, VIII; between Group II and Groups III, VI (resp., *P* = 0.009, 0.034, 0.017, and 0.024). In Group VIII (8 rats in total), 2 rats showed grade 3 and 3 showed grade 2; the other 3 rats showed grade 1 inflammations. In Group III (9 rats in total), 1 rat showed grade 2 and 8 showed grade 1 inflammations. In Group VI (8 rats in total), 1 rat showed grade 2 and 7 showed grade 1 inflammations (Figure 1).

Oligonol and oligonol plus quantum groups were showed to have stronger anti-inflammatory effects than in the other groups (Table 3).

## 4. Discussion

Injection of E2 into rats results in prostatic inflammation. Whereas combination of E2 with testosterone prevents tissue atrophy and inflammation, DHT allows the persistence of E2-induced inflammation while preventing tissue atrophy [34]. In this study, E2 and DHT (250 micrograms/kg/day) doses proposed by Naslund et al. [4] were used to induce chronic prostatitis (CP).

We have to use the suitable doses of oligonol, estradiol, and DHT that would be affected by body weight changes in this experimental model. Kim and colleagues [1] mentioned that the estradiol + DHT and oligonol treated groups showed significantly reduced prostate weight compared to the normal control group (*P* < 0.001). Additionally, no significant difference in prostate weight was noted between the estradiol + DHT and oligonol treated groups (*P* = 0.125).

In oligonol groups, significantly fewer increases in body weight at day 40 were found as compared to the normal control group. CP and other treatment groups showed significantly reduced prostate weight compared to the normal control group. The reduced prostate weight was most likely due to decreased secretion production. E2 given with DHT could restore wet prostate weight which does not include the secretion weight and could decrease secretion production as well [1]. The 4-point inflammation grading system, used by Bernoulli and colleagues, was applied in evaluating the severity of inflammation of lateral prostate lobes [35]. The severity of inflammation was assessed according to the aggressiveness of inflammation and by counting the number of inflamed acini from grade 0 to grade 3. Wistar rats represent appropriate animal models for experimental CP studies since aging is associated with spontaneous development of nonbacterial prostatitis in these species [3]. So, median prostatitis score for the control group was 1 with a range between 0 and 2. On microscopic examinations, the oligonol and oligonol plus quantum groups showed an effectively reduced inflammation of the prostate and degeneration of the glandular epithelium compared to the other treatment groups. It has been proposed that oligonol shows beneficial effects in the treatment of nonbacterial CP through the regulation of antioxidative mechanisms, proinflammatory cytokines, and IKBa phosphorylation [1]. Antioxidant effects of oligonol have been previously demonstrated in a number of studies [1, 3, 36]. In a study by Kim et al. [1], an association between the development of nonbacterial prostatitis and significantly reduced activity of superoxide dismutase (SOD) and glutathione peroxidase (GPx) in the prostate tissue has been shown. The same authors have also reported a greater effect of oligonol on GPx activation. The marked similarity between human CP and E2-induced CP in rats may help explain beneficial therapeutic and preventive effects of oligonol on patients with CP/CPPS IIIA [1].

As previously proposed by Vladimirov et al. [37], experimental evidence exists to explain the stimulatory effects of the low intensity laser and noncoherent beams with free radical mechanisms. They have also pointed out a proven association between the primary mechanisms of the stimulatory effects of light and the secondary mechanisms defining the sanitation effects (bactericidal effect, cellular proliferation, and regulation of microcirculation). In addition, laser beams used for the quantum therapy have been reported to induce an ordered series of nonspecific regulatory reactions with revitalizing, rejuvenating, anesthetic, and antiphlogistic effects [12]. For example, Kogan and colleagues have reported significant superiority of magnetolaser over standard pharmaceutical treatments in terms of the improvement in pain, urinary symptoms, and quality of life scores in the treatment of inflammatory chronic nonbacterial prostatitis [38] that are in line with these research experimental findings. We can say that quantum light therapy is a new approach to CP's treatment as an inexpensive and reasonable alternative treatment that has no apparent side effects.

A good body of evidence suggests that acupuncture therapy helps with anti-inflammatory and analgesic effects [39, 40]. In addition, a report has proposed that the pain in CP/CPPS may be of neuropathic character and acupuncture has neuromodulatory effects [41]. In this study, the acupuncture points most frequently preferred for the treatment of CP/CPPS, that is, CV 3 and 4 bilaterally B1 32 and 34, were used [39] under the guidance of a Rat Atlas and Acupuncture Atlas [32, 33] by an acupuncturist. The mechanism for the anti-inflammatory effect of acupuncture is not yet clear. However, in this study, when the differences between Groups III, VI, VII, and IX were compared, the acupuncture group showed a less anti-inflammatory effect than the other groups. On the other hand, the anti-inflammatory effects of acupuncture were thought to be less effective than oligonol and quantum light. A better median prostatitis score in oligonol + quantum group was obtained, but, due to the limited number of animals in these groups, a statistically significant difference may not be visible.

The mechanisms of anti-inflammatory effects on acupuncture are not yet clear. However, a significant interpersonal variability in the levels of electroacupuncture (EA) analgesia has previously been reported both in animals and in humans [22, 23]. Proposed mechanisms for the effect of EA include the release of endogenous opioid peptides and activation of descendant inhibitory pathways of the CNS [42]. However, EA was not used in this study and manual stimulation of the needles with 10-minute intervals was performed.

## 5. Conclusions

Although all of the treatment modalities tested in this study showed anti-inflammatory effects in the treatment of estrogen-induced chronic nonbacterial prostatitis in male rats, a better median prostatitis score in oligonol plus quantum light group and oligonol group was obtained. Monotherapy with oligonol showed a superior anti-inflammatory efficacy as compared to quantum light and acupuncture monotherapies. But it might be thought that the combination therapy such as oligonol plus quantum light has a synergism. We believe that findings of this study may help further stimulate multicenter prospective studies that would better quantify these findings.

## Figures and Tables

**Table 1:** Structure of the experiment.

Groups		*n*	Drug treatment	Agents administrated to induce inflammation
Group I	Normal control	10	—	—
Group II	Chronic prostatitis	10	—	E2 0.25 mg/kg (s.c.) + DHT 0.25 mg/kg (s.c.)
Group III	Oligonol	9	Oligonol 60 mg/kg (p.o.)	10 E2 0.25 mg/kg (s.c.) + DHT 0.25 mg/kg (s.c.)
Group IV	Acupuncture	7	—	10 E2 0.25 mg/kg (s.c.) + DHT 0.25 mg/kg (s.c.)
Group V	Quantum	7	—	10 E2 0.25 mg/kg (s.c.) + DHT 0.25 mg/kg (s.c.)
Group VI	Oligonol + quantum	8	Oligonol 60 mg/kg (p.o.)	10 E2 0.25 mg/kg (s.c.) + DHT 0.25 mg/kg (s.c.)
Group VII	Acupuncture + oligonol	9	Oligonol 60 mg/kg (p.o.)	10 E2 0.25 mg/kg (s.c.) + DHT 0.25 mg/kg (s.c.)
Group VIII	Quantum + acupuncture	8	—	10 E2 0.25 mg/kg (s.c.) + DHT 0.25 mg/kg (s.c.)
Group IX	Quantum + oligonol + acupuncture	9	Oligonol 60 mg/kg (p.o.)	10 E2 0.25 mg/kg (s.c.) + DHT 0.25 mg/kg (s.c.)

E2, 17-beta-estradiol; DHT, dihydrotestosterone; s.c., subcutaneous injection; p.o., oral administration.

**Table 2:** Parameters of the quantum light therapy used.

Wavelength of impulsive infrared laser radiation: 890–910 nanometers	
Wavelength of pulsating broadband infrared radiation: 860–960 nanometers	
Wavelength of pulsating broadband red radiation: 40–740 nanometers	
Frequency setting: 50 Hz	
Frequency of red light radiation: 2 Hz	
Magnetic induction: 35 ± 10 mTl	
Time of radiation: 5 min.	
Power supply: alternating current	
Frequency: 50/60 Hz	
Power consumed from an electric network: 20 W	
Power: 12 W emitter and 30 W emitter (perineum)	
Beam area at the skin: 4 cm^2^	
Anatomical location: suprapubic, urinary bladder, anterior penile, and femoral artery (bilateral)	
regions and mid-perineum region between testicles and anus (including prostate area)	
Number of treatments: 18 procedures	
Interval between treatments: 3 days a week for a total duration of 6 weeks	

**Table 3:** Body weight, prostate weight, and prostatitis score of the groups.

		BW day 1 (gr)	BW day 20 (gr)	BW day 40 (gr)	Prostate weight (mg)	Prostatitis score
Groups		Mean ± SD	Mean ± SD	Mean ± SD	Mean ± SD	Median (min–max)
		Median (min–max)	Median (min–max)	Median (min–max)	Median (min–max)	
Group I	Normal control (*n*: 10)	314.40 ± 20.57	371.00 ± 29.06	412.60 ± 16.44	1,046.80 ± 61.08	1 (0–2)
		312.50 (289–350)	364.00 (325–408)	419.50 (380–430)	1,030.50 (979–1130)	

Group II	Chronic prostatitis (*n* : 10)	311.80 ± 15.29	345.20 ± 27.98	367.80 ± 35.89	950.00 ± 25.98	2 (1–3)
		317.00 (287–330)	342.50 (315–392)	373.00 (321–420)	950.50 (920–985)	

Group III	Oligonol (*n*: 9)	319.67 ± 10.44	362.78 ± 23.02	380.33 ± 14.47	964.00 ± 17.41	1 (1-2)
		324.00 (302–330)	373.00 (322–378)	385.00 (354–394)	974.00 (939–980)	

Group IV	Acupuncture (*n*: 7)	316.86 ± 15.04	372.43 ± 23.41	406.71 ± 14.04	947.86 ± 28.75	2 (1-2)
		321.00 (294–332)	381.00 (335–394)	409.00 (390–425)	934.00 (926–992)	

Group V	Quantum (*n*: 7)	310.00 ± 14.00	341.86 ± 29.01	381.43 ± 25.25	942.71 ± 2.09	1 (1–3)
		308.00 (294–331)	326.00 (321–389)	384.00 (350–417)	935.00 (928–991)	

Group VI	Oligonol + quantum (*n*: 8)	313.50 ± 9.77	344.12 ± 19.74	383.88 ± 14.15	950.25 ± 19.61	1 (1-2)
		314.00 (298–325)	343.00 (323–386)	388.00 (368–417)	945.00 (928–975)	

Group VII	Acupuncture + oligonol (*n*: 9)	311.22 ± 8.87	349.22 ± 27.79	380.11 ± 13.82	940.78 ± 8.03	1 (1–3)
		309.00 (301–326)	331.00 (323–387)	372.00 (364–399)	940.00 (930–953)	

Group VIII	Quantum + acupuncture (*n*: 8)	308.88 ± 9.43	358.75 ± 27.35	386.00 ± 21.82	956.38 ± 25.15	2 (1–3)
		307.50 (294–321)	356.00 (329–392)	380.50 (358–416)	939.50 (935–989)	

Group IX	Quantum + oligonol + acupuncture	312.44 ± 14.40	367.67 ± 32.35	388.33 ± 15.50	953.56 ± 17.27	1 (1–3)

SD: standard deviation; median (min–max): (minimum–maximum) values; BW: body weight; gr: gram; mg: milligram.
